# Experimental Researches on the Action of Caustic Potassa

**Published:** 1860-03

**Authors:** 

**Affiliations:** Surgeon of Hôtel-Dieu, at Chartres; Surgeon of Hôtel-Dieu, at Chartres


*Experimental Researches on the Action of Caustic Potassa.* By SALMON and MAUNOURY, Surgeons of Hôtel-Dieu, at Chartres.

The authors present the contradictory views of numerous surgical writers as to the action of caustic potassa, showing that they vary: 1st, as to the rapidity of the action of potassa in producing eschars—some indicating twenty minutes as sufficient, and others nearly twelve hours; 2d, as to the possibility of regulating its penetrative properties—some opposing the dilution of the agent, and others considering that it is best; 3d, as to the pain produced, Canquoin claiming that it is quite supportable, &c.; 4th, as to the hæmorrhagic tendency: Giroward advising that the tissues should be plentifully washed with water when one operates over vessels of medium size; Canquoin, that this always exposes to sudden hæmorrhages, &c. These questions are attempted to be solved by the experiments made upon animals in 1851, on the human body, and on dead bodies, and the following conclusions are arrived at:

1. Caustic potassa is an agent which very rapidly effects cauterization.

2. It may convert the whole thickness of the skin, covered with epidermis, into an eschar in fifteen minutes; it can perforate a voluminous muscular mass in from six to ten minutes; it produces a hole in cellular and fungous tissues directly; it rapidly destroys vessels, has a slower action upon fibrous tissues, and does not dissolve osseous tissues.

3. It acts upon the tissues by dissolving them. The eschar presents an appearance as if the skin were cut out by a punch; under the action of potassa muscular fibres and the coats of the vessels become thin, tight, dissolve, or finally burst open. Blood touched by it coagulates at first, its course being arrested; it then becomes liquid, and finally flows. The cornea may be pierced by it, and ligaments and cartilages may be slowly destroyed.

4. Despite its activity in destruction, its action may be limited; on the skin we may make a linear incision, so as to prevent the solution of potassa spreading beyond the limits we wish preserved; below the surface, cellular tissue excepted, the action of potassa circumscribes itself, either by the amount required to dissolve the eschar produced, or by dilution in the liquids, or by forming non-caustic compounds with the tissues.

5. Potassa rapidly effects the destruction of vessels, and facilitates hæmorrhages; but its first effect is to coagulate the blood, and as this coagulum separated from the action of potassa becomes very hard, it is easy to avoid the dangers from hæmorrhage; when the action of the potassa in producing the coagulum is accomplished, the caustic must be removed, so that the after solution is not produced. This can be done by using a *tampon* immediately, made either of dry cotton, or of cotton saturated with vinegar, or with slips of Canquoin's caustic. Potassa may thus be employed to check hæmorrhages, through the rapid formation of a coagulum.

6. Potassa can dissolve the eschars it makes, although it is easier, in the human subject, to remove them by incision. The incision should be made while the eschar is fresh, as it speedily becomes very hard.—*Gazette Médicale.* L. H. S.

